# Euglycemic Ketoacidosis Induced by Dapagliflozin in a Non‐Diabetic Patient With Cardiac Amyloidosis

**DOI:** 10.1002/ccr3.71818

**Published:** 2026-01-07

**Authors:** Salhamoud Abdelfatah Saleh Hamoud, Ellie Harding

**Affiliations:** ^1^ Department of Medicine University Hospitals Plymouth NHS Trust Plymouth UK

**Keywords:** cardiac amyloidosis, dapagliflozin, euglycemic ketoacidosis, non‐diabetic patient, SGLT2 inhibitors

## Abstract

Dapagliflozin, though beneficial in heart failure, can rarely cause euglycemic ketoacidosis even in non‐diabetic patients. Clinicians should maintain a high index of suspicion for metabolic acidosis in patients on SGLT2 inhibitors presenting with unexplained illness, regardless of blood glucose level.

## 1 Introduction

Sodium–glucose co‐transporter 2 (SGLT2) inhibitors, including dapagliflozin, canagliflozin, and empagliflozin, have proven benefits in reducing morbidity and mortality in heart failure, regardless of diabetes status [1, 2]. However, their use carries a risk of euglycemic ketoacidosis (eKA), characterized by significant ketosis and metabolic acidosis without marked hyperglycemia [3, 4, 5]. Although well described in diabetic populations, reports in non‐diabetics remain scarce [6, 7].

Cardiac amyloidosis is a restrictive cardiomyopathy caused by extracellular amyloid fibril deposition, often involving multiple organ systems including the gastrointestinal tract and kidneys [8, 9]. These effects may predispose to dehydration, reduced oral intake, and autonomic dysfunction, potentially increasing the risk of metabolic complications related to SGLT2 inhibitors.

## 2 Case History and Examination

An 88‐year‐old man with ischaemic heart disease, chronic heart failure (LVEF 45%), moderate mitral regurgitation, pulmonary artery systolic pressure 58 mmHg, and transthyretin cardiac amyloidosis treated with tafamidis (61 mg daily) presented with generalized weakness and confusion.

His daughter reported progressive fatigue and reduced oral intake over several weeks, followed by 4 days of diarrhea and vomiting. On the day of admission, he developed upper abdominal discomfort and acute confusion. Due to his confusion, a reliable history regarding alcohol consumption could not be obtained.

He had no history of diabetes. His current medications included dapagliflozin 10 mg daily (for 5 months), bumetanide, nicorandil, bisoprolol, apixaban, ranolazine, and tafamidis.

On examination, he was afebrile, with a blood pressure of 106/60 mmHg, a heart rate of 105 bpm (irregularly irregular), and a respiratory rate of 22 breaths per minute. Oxygen saturation was 96% on room air. He appeared dehydrated with dry mucous membranes and reduced skin turgor. Jugular venous pressure was not elevated. Cardiovascular examination revealed an irregular rhythm with a soft systolic murmur at the apex. Abdominal examination was soft with mild epigastric tenderness. No focal neurological deficit was found. ECG revealed new‐onset atrial fibrillation without ST‐T changes.

Urine culture grew *Enterococcus faecalis* sensitive to co‐amoxiclav. HbA1c was 43 mmol/mol (normal range < 42 mmol/mol). Echocardiography showed concentric LV hypertrophy with apical trabeculations consistent with cardiac amyloidosis. His blood tests on admission are summarized in Table 1.

**TABLE 1:** Key investigations.

Parameter	Result	Reference range
pH	7.16	7.35–7.45
pCO_2_ (kPa)	5.10	4.7–6.0
Bicarbonate (mmol/L)	14.1	22–28
Lactate (mmol/L)	4.3	0.5–2.2
Glucose (mmol/L)	10.8	3.9–7.8
Serum ketones (mmol/L)	3.2	< 0.6
Anion gap (mmol/L)	45.6	8–16
Creatinine (μmol/L)	141	64–104
Sodium (mmol/L)	137	133–146
Potassium (mmol/L)	5.5	3.5–5.3
eGFR (mL/min/1.73m^2^)	38	> 60
Urea (mmol/L)	21.1	2.5–7.8
Chloride (mmol/L)	111	98–107
Bilirubin (μmol/L)	9	1–20
Alanine transaminase (IU/L)	45	1–55
Alkaline phosphatase (IU/L)	80	30–130

## 3 Differential Diagnosis, Investigations, and Treatment

Differential diagnoses included the following:

- eKA secondary to dapagliflozin.
- Starvation ketoacidosis due to poor intake.
- Lactic acidosis from hypoperfusion.
- Sepsis‐related metabolic acidosis from urinary tract infection.
- Mixed metabolic acidosis from multifactorial causes.

The diagnosis of eKA was reached based on the triad of metabolic acidosis (high anion gap, low bicarbonate), significant ketonemia, and euglycemia in a patient taking an SGLT2 inhibitor. While sepsis, dehydration, and poor intake were significant contributors, the profound ketosis (3.2 mmol/L) with only modest hyperglycemia is characteristic of SGLT2 inhibitor‐induced eKA. Starvation ketoacidosis alone was unlikely to cause such severe acidosis, and lactic acidosis, while present, did not fully account for the high anion gap.

*Treatment*: The patient was managed per the hospital DKA protocol with intravenous fluid resuscitation (0.9% saline and 10% dextrose infusion) and a fixed‐rate intravenous insulin infusion starting at 6.6 U/h (0.1 Unit/kg body weight/h). Table 2 summarizes the key serial metabolic parameters during treatment. Ketones normalized to 0.8 mmol/L within 2 h. Hypoglycemia (glucose 2.7 mmol/L) developed 6 h into treatment, prompting a switch to a variable‐rate insulin infusion with 10% dextrose. The metabolic acidosis resolved (pH 7.33, bicarbonate 16.1 mmol/L) within 12 h. Dapagliflozin was discontinued permanently [1, 2]. Co‐amoxiclav was started for 7 days for the urinary tract infection.

**TABLE 2:** Serial metabolic parameters during treatment—glucose, ketones, and insulin response.

Date	Time	Glucose (mmol/L)	Ketones (mmol/L)	Potassium (mmol/L)	Bicarbonate/pH
23/07/25	15:51	10.9	3.2	4.3	14.9/7.22
23/07/25	16:56	10.2	—	5.0	13.8/7.17
23/07/25	17:56	11.3	0.8	—	—
23/07/25	20:05	7.0	1.1	4.3	14.2/7.20
23/07/25	21:13	7.2	0.8	—	—
24/07/25	00:20	2.7	0.1	3.8	14.9/7.28
24/07/25	05:41	6.9	—	4.1	16.1/7.33

## 4 Outcome and Follow‐Up

The patient made a full metabolic recovery and resumed oral intake. He was discharged in stable condition, with dapagliflozin discontinued and sick‐day rules reinforced.

## 5 Discussion

This case illustrates that SGLT2 inhibitors can precipitate eKA in non‐diabetic patients, especially in the presence of acute illness, dehydration, infection, and multi‐organ involvement [3, 4, 5, 6, 7]. SGLT2 inhibitors promote glycosuria and impair glucose reabsorption, leading to lower blood glucose. Concurrently, they stimulate glucagon secretion, enhance lipolysis, and promote hepatic ketogenesis [3, 4]. In a metabolically stressed state (e.g., from infection, dehydration), insulin secretion may be suppressed, further tipping the balance toward ketogenesis. It is important to note that while the term “euglycemic DKA” is widely used, recent consensus [7] specifies DKA typically occurs in individuals with diabetes. As our patient was non‐diabetic, his presentation is more accurately described as SGLT2 inhibitor‐induced euglycemic ketosis, a critical differential for metabolic acidosis that falls outside traditional diagnostic guidelines. In this patient, prolonged diarrhea, vomiting, and a urinary tract infection created a perfect metabolic storm. His advanced age, associated with an age‐related decline in beta‐cell function, may have further reduced his metabolic reserve. Cardiac amyloidosis likely contributed via gastrointestinal dysmotility, malabsorption, and autonomic dysfunction, increasing susceptibility to dehydration and impaired metabolic adaptation [8, 9, 10].

The initial high‐dose insulin infusion, based on a standard DKA protocol, led to rapid ketone clearance but also hypoglycemia. This highlights a key management difference in SGLT2 inhibitor‐induced eKA in non‐diabetics. These patients have preserved endogenous insulin secretion and sensitivity. Therefore, the ketoacidosis is often exquisitely sensitive to therapy, and vigorous fluid resuscitation alone or with very low‐dose insulin (e.g., 0.05 U/kg/h) is frequently sufficient, minimizing hypoglycemia risk [7, 11]. The profound ketosis in our patient resolved quickly, but the standard insulin dose proved excessive, underscoring the need for a tailored approach. Bicarbonate correction typically lags behind pH improvement during treatment of ketoacidosis due to normal physiological mechanisms. Once insulin suppresses ketogenesis, pH rises quickly as respiratory compensation decreases and pCO_2_ normalizes. However, bicarbonate regenerates more slowly because it depends on hepatic metabolism of ketoacids, renal recovery of bicarbonate reabsorption, and resolution of a transient hyperchloremic acidosis caused by saline resuscitation [12]. This delayed bicarbonate rise is expected and does not indicate treatment failure.

As heart failure guidelines now endorse SGLT2 inhibitors in non‐diabetic patients [1, 2], awareness of this rare but potentially life‐threatening complication is vital.

## 6 Search Strategy

A literature search (PubMed, Embase, and Google Scholar; up to August 2025) using combinations of the keywords ‘euglycaemic ketoacidosis’, ‘euDKA’, ‘SGLT2 inhibitors’, ‘dapagliflozin’, ‘non‐diabetic,’ and ‘cardiac amyloidosis’ revealed multiple euDKA reports in diabetic patients [3, 4, 5, 6], a small number in non‐diabetics [2, 6], but no previous reports linking dapagliflozin‐induced eKA with cardiac amyloidosis.

## 7 How This Report Adds to the Literature

- Novel association: First report of dapagliflozin‐induced euglycemic ketosis in a non‐diabetic patient with transthyretin amyloidosis.
- Risk factors: Highlights how systemic amyloidosis may increase vulnerability to metabolic decompensation [8, 9, 10].
- Clinical implication: Reinforces the critical need for sick‐day guidance, close monitoring, and consideration of euglycemic ketosis during intercurrent illness in all patients on SGLT2 inhibitors [1, 2].

## 8 Learning Points

1. eKA can occur in non‐diabetic patients treated with SGLT2 inhibitors.
2. A high index of suspicion is required as the presentation can be masked by normal or only slightly elevated blood glucose.
3. Management should prioritize fluid resuscitation with low dose insulin (0.05 U/KG/h) due to insulin sensitivity.
4. Cardiac amyloidosis with its multi‐system involvement may increase risk through systemic and metabolic vulnerability.
5. Sick‐day rules should be emphasized for all patients on SGLT2 inhibitors.

## Author Contributions

**Salhamoud Abdelfatah Saleh Hamoud:** conceptualization, supervision, writing – original draft, writing – review and editing. **Ellie Harding:** data curation, resources, writing – original draft.

## Funding

The authors have nothing to report.

## Consent

Written informed consent for publication was obtained from the patient.

## Data Availability

The data that support the findings of this study are available within the article.
